# Partial inhibition of the ubiquitin–proteasome system ameliorates cardiac dysfunction following ischemia–reperfusion in the presence of high glucose

**DOI:** 10.1186/s12933-015-0258-4

**Published:** 2015-07-28

**Authors:** Buin Adams, Rudo F Mapanga, M Faadiel Essop

**Affiliations:** Cardio-Metabolic Research Group (CMRG), Department of Physiological Sciences, Stellenbosch University, Room 2005, Mike De Vries Building, Merriman Avenue, Stellenbosch, 7600 South Africa

**Keywords:** Ubiquitin–proteasome system, Ischemia–reperfusion, Cardiac dysfunction, Hyperglycemia, Inflammation, Oxidative stress, Autophagy

## Abstract

**Background:**

Acute hyperglycemia co-presenting with myocardial infarction (in diabetic and non-diabetic individuals) is often associated with a poor prognosis. Although acute hyperglycemia induces oxidative stress that can lead to dysregulation of the ubiquitin–proteasome system (UPS), it is unclear whether increased/decreased UPS is detrimental with ischemia–reperfusion under such conditions. As our earlier data implicated the UPS in cardiac damage, we hypothesized that its inhibition results in cardioprotection with ischemia–reperfusion performed under conditions that simulate acute hyperglycemia.

**Methods:**

Ex vivo rat heart perfusions were performed with Krebs–Henseleit buffer containing 33 mM glucose vs. controls (11 mM glucose) for 60 min stabilization, followed by 20 min global ischemia and 60 min reperfusion ± 5 µM lactacystin and 10 µM MG-132, respectively. The UPS inhibitors were added during the first 20 min of the reperfusion phase and various cardiac functional parameters evaluated. In parallel experiments, infarct sizes were assessed following 20 min regional ischemia and 120 min reperfusion ± each of the respective UPS inhibitors (added during reperfusion). Heart tissues were collected and analyzed for markers of oxidative stress, UPS activation, inflammation and autophagy.

**Results:**

The proteasome inhibitor doses and treatment duration here employed resulted in partial UPS inhibition during the reperfusion phase. Both lactacystin and MG-132 administration resulted in cardioprotection in our experimental system, with MG-132 showing a greater effect. The proteasome inhibitors also enhanced cardiac superoxide dismutase protein levels (SOD1, SOD2), attenuated pro-inflammatory effects and caused an upregulation of autophagic markers.

**Conclusions:**

This study established that partial proteasome inhibition elicits cardioprotection in hearts exposed to ischemia–reperfusion with acute simulated hyperglycemia. These data reveal that protease inhibition triggered three major protective effects, i.e. (a) enhancing myocardial anti-oxidant defenses, (b) attenuating inflammation, and (c) increasing the autophagic response. Thus the UPS emerges as a unique therapeutic target for the treatment of ischemic heart disease under such conditions.

## Background

The link between hyperglycemia and increased morbidity/mortality with heart diseases such as acute myocardial infarction (AMI) is receiving growing attention [1–3]. Hyperglycemia can manifest as a more chronic condition (typically found with diabetes) or as a stress-induced state, e.g. in diabetic and non-diabetic persons challenged by an AMI. Chronic hyperglycemia may sometimes be linked to the development of macrovascular complications [4], with AMI the chief contributor to cardiovascular mortality with diabetes [5–7]. Acute, stress-induced hyperglycemia is associated with increased in-hospital deaths, congestive heart failure, and cardiogenic shock [8–10]. The presentation of hyperglycemia in non-diabetic individuals likely represents a combination of undiagnosed diabetes, impaired glucose tolerance [11, 12], and a robust response to acute stress [8, 9].

Hyperglycemia-induced oxidative stress can lead to the formation of misfolded or damaged proteins that are usually eliminated by the ubiquitin–proteasome system (UPS), the major role player responsible for non-lysosomal protein degradation [13, 14]. However, a dysfunctional UPS is linked with a pro-inflammatory milieu and impaired cardiac function in diabetic mice [15] and diabetic patients presenting with unstable angina [16]. Although altered UPS functioning is implicated with diminished cardiac function, it remains unclear whether greater or lesser UPS activation is detrimental with ischemia–reperfusion under such conditions. This conundrum also applies for UPS regulation in response to ischemia–reperfusion per se, i.e. under normoglycemic conditions. For example, some found that oxidative stress, inflammation and myocardial reperfusion injury can be blunted by proteasomal inhibitor treatment [15, 16]. Furthermore, UPS inhibition leads to a reduction in ventricular tachyarrhythmias [17] and preserved cardiac function following myocardial ischemia under baseline glucose conditions. Conversely, others established that UPS inactivation (due to oxidative damage occurring with ischemia–reperfusion) is a robust contributor to the damaging effects elicited by an AMI [18]. Such discordant findings may be due to different experimental protocols/models utilized and due to variations in UPS inhibitor class, dosages and length of administration [19, 20]. With the paucity of studies investigating UPS regulation with ischemia–reperfusion under acute hyperglycemic conditions, efforts to gain greater insight into this intriguing question are essential as it will contribute to (a) the delineation of underlying mechanisms, and (b) the development of novel cardioprotective agents.

As our earlier findings implicated increased UPS activation in hyperglycemia-mediated cardiac damage [21] we hypothesized that its inhibition during early reperfusion provides cardioprotection following ischemia–reperfusion under high glucose conditions. We employed an established rat model of ex vivo isolated heart perfusions subjected to ischemia–reperfusion and exposed to simulated acute hyperglycemia as before [21, 22]. To further strengthen these data two different UPS inhibitor classes were used, i.e. lactacystin (2-(acetylamino)-3-[({3-hydroxy-2-[1-hydroxy-2-methylpropyl]-4-methyl-5-oxopyrrolidin-2-yl}carbonyl)sulfanyl]propanoic acid) and MG-132 (*N*-(benzyloxycarbonyl) leucinylleucinylleucinal Z-Leu-Leu-Leu-al). The current study demonstrates that proteasome inhibition elicits cardioprotection in hearts exposed to ischemia–reperfusion with acute simulated hyperglycemia by upregulating anti-oxidant defenses, enhancing anti-inflammatory effects and increasing autophagy.

## Methods

### Animals and ethics statement

All animals were treated in accordance with the Guide for the Care and Use of Laboratory Animals of the National Academy of Sciences (NIH publication No. 85-23, revised 1996). This study was carried out with the approval of the Animal Ethics Committee of Stellenbosch University.

### Ex vivo perfusion protocol

Male Wistar rats weighing 180–220 g were used for this study. Rats were anesthetized (pentobarbitone, 100 mg/kg i.p.) and hearts rapidly excised and perfused in a modified Langendorff model as described before [21, 22]. Briefly, Krebs–Henseleit buffer containing (in mM) 11 glucose, 118 NaCl, 4.7 KCl, 1.2 MgSO_4_·7H_2_O, 2.5 CaCl_2_·2H_2_O, 1.2 KH_2_PO_4_, 25 NaHCO_3_ was equilibrated with 95% O_2_–5% CO_2_ (37°C, pH 7.4) at a constant pressure (100 cm). Buffer was not recirculated and the hearts were allowed to beat at their natural rate. During perfusion, a latex balloon attached to a pressure transducer (Stratham MLT 0380/D, ADInstruments Inc, Bella Vista, NSW, Australia) compatible with the PowerLab System ML410/W (ADInstruments Inc, Bella Vista, NSW, Australia) was inserted into the left ventricle via the mitral valve and inflated to produce a systolic pressure of 80–120 mmHg and a diastolic pressure of 4–12 mmHg. The temperature of the heart was maintained at 37°C by suspending it in a heated water jacket.

The protocol was divided into two parts, i.e. perfusions (a) with global ischemia and (b) regional ischemia, followed by reperfusion for cardiac functional assessments and infarct size determination, respectively. For each of these, hearts were randomly distributed into six experimental groups for perfusions: baseline control (11 mM glucose) ± either 5 μM lactacystin or 10 μM MG-132; and high glucose (33 mM glucose) ± either 5 μM lactacystin or 10 μM MG-132 (n = 8 rats were used for each of the experimental groups indicated). The respective UPS inhibitors (lactacystin, MG-132) were dissolved in the buffer and added for the first 20 min of reperfusion.

For our inhibition studies we aimed to achieve so-called “non-toxic proteasome inhibition’’, i.e. only partial attenuation of chymotrypsin-like activity as high and sustained doses can result in severe toxic effects with detrimental outcomes [23]. Lactacystin is a natural compound that binds mainly to the β5 proteasomal subunit (responsible for chymotrypsin-like activity) of the 20S proteasome [24], leading to irreversible inhibition of chymotrypsin-like activity (reviewed in [25]). However, the trypsin- and caspase-like activities are blunted to a lesser extent in this instance [26]. We also employed the reversible aldehyde inhibitor MG-132 that specifically binds to proteasomal subunit β5 of the 20S proteasome (reviewed in [25]). Proteasome inhibitor doses were selected to be in the range of what was employed in previously published studies [27, 28]. High glucose perfusions were used to simulate acute hyperglycemia and since ex vivo Langendorff perfusions are typically performed with 11 mM glucose at baseline, the 33 mM dose would be representative of a threefold elevation of glucose levels (above normal) within the clinical setting. Additional experiments were also performed in order to rule out the effects of osmotic pressure on heart function. Here rat hearts were perfused with 22 mM mannitol plus 11 mM glucose (total molarity = 33 mM) and subjected to ischemia–reperfusion as described above.

### Ex-vivo global ischemia and reperfusion

The protocol included a 60 min stabilization period, 20 min of global ischemia, followed by 60 min of reperfusion. Contractile parameters assessed throughout the experiment included heart rate (HR), left ventricular developed pressure (LVDP), end diastolic pressure (EDP), rate-pressure product (RPP = HR × LVDP), and coronary flow. The percentage recovery for LVDP and RPP was also calculated where reperfusion data points were expressed as a percentage of pre-ischemic values. Coronary flow was measured by collection of the effluent at regular timed intervals. Both left and right ventricular tissues were immediately collected after the 60 min reperfusion period with tissues freeze-clamped in liquid nitrogen with pre-cooled Wollenberger tongs, whereafter it was stored at −80°C for further molecular and biochemical analyses.

### Ex-vivo regional ischemia and reperfusion

To further strengthen our Langendorff functional data, we also evaluated the effects of lactacystin and MG-132 by infarct size determination. This was performed as described before [21, 22], and we employed regional ischemia with a reperfusion time of 2 h. Here a 3/0 silk suture was placed on the proximal portion of the left anterior descending coronary artery and passing the ends through a plastic tube. For induction of regional ischemia the ends were tightened by pressing the plastic tube against the surface of the heart (above the artery) for 20 min. The snare was released during the reperfusion period. The efficacy of ischemia was confirmed by regional cyanosis and a substantial decrease in coronary flow.

After completion of each regional ischemia–reperfusion experiment the snare was re-tightened and 2.5% Evans blue dye (in Krebs buffer) was perfused through the hearts for identification of the area at risk of ischemia. Hearts were subsequently removed from the Langendorff apparatus, blotted dry, suspended (using suture) within 50 ml plastic tubes and frozen at −20°C for 3 days. Frozen hearts were thereafter sliced into 2 mm transverse sections and incubated with 1% 2,3,5-triphenyl tetrazolium chloride in phosphate-buffered saline for 20 min at 37°C. Slices were then fixed in 10% formalin for 24 h at room temperature before being placed between glass plates for scanning. The infarct size and the area-at-risk were determined using Image J software (v1.46p, NIH, USA) and infarct size was expressed as a percentage of the area-at-risk.

### Measurement of intracellular E3 ligases, oxidative stress, inflammation and autophagic markers

Protein isolation was performed as previously described [29]. Briefly, collected heart tissues were homogenized with modified RIPA buffer, the supernatant centrifuged twice at 4,300*g* for 10 min at 4°C then stored at −80°C until further use. Protein expression was determined by SDS-PAGE as described previously by our laboratory [21, 29], and Western blotting performed with representative markers for: (a) E3 ligases; Muscle RING Finger 1 (MURF1; Abcam, Cambridge, MA, USA) and muscle atrophy F-box (MAFbx; Santa Cruz Biotechnologies, Santa Cruz, CA, USA); (b) oxidative stress: superoxide dismutase 1 and 2 (SOD1, SOD2; Santa Cruz Biotechnologies, Santa Cruz, CA, USA); (c) inflammation: tumor necrosis factor-alpha (TNF-α; Sigma-Aldrich, St. Louis, MO, USA), and inhibitor of nuclear factor kappa-B kinase subunit alpha (IκBα; Sigma-Aldrich, St. Louis, MO, USA); and (d) autophagy: microtubule‐associated protein 1 light chain 3 II (LC3-II; Cell Signaling, Danvers, MA, USA), and p62 (Cell Signaling, Danvers, MA, USA). Western blots were quantified by densitometric analysis and β-actin (Cell Signaling, Danvers, MA, USA) employed as a loading control as described before [21, 22].

### Proteasome activity experiments

Heart tissues were cut into small slices and homogenized in 1 ml of Tris–HCl buffer (pH 7.4) using an IKA Ultra Turrax T25 homogenizer (IKA Labortechnik, Staufen, Germany) and incubated on ice for 10 min before centrifugation at 9,000*g* for 15 min to remove cell debris. The supernatant was employed for protein quantification using the Bradford assay as described previously [30].

The Proteasome-Glo™ 3-Substrate System (Promega, Madison, WI, USA) consists of three homogeneous bioluminescent assays that separately measure the three proteolytic activities associated with the proteasome, i.e. chymotrypsin-like (LLVY), trypsin-like (LSTR), and caspase-like (LLE) activities. The three assays differ in their ability to detect different protease activities based on their substrate components, i.e. the luminogenic substrates provided for the LLVY, LSTR, and LLE are Suc-LLVY-aminoluciferin, Z-LRR-aminoluciferin, and Z-nLPnLD-aminoluciferin, respectively. Each substrate is added to a buffer system optimized for proteasome and luciferase activities to make a Proteasome-Glo™ Reagent for a particular catalytic activity. The individual Proteasome-Glo™ Reagent is added to test samples in an “add-mix-measure” format, resulting in proteasome-induced cleavage of each particular substrate. Substrate cleavage generates a “glow-type” luminescent signal produced by the luciferase reaction that is proportional to proteasome activity.

Assays were performed with ~50 µg of protein lysate [in 25 mM Tris–HCl (pH 7.5)] together with the respective substrate—incubated together for 30 min at 37°C. Aminomethylcoumarin and β-naphthylamine luminescence was subsequently measured using a luminometer microplate reader (BMG Labtech, Ortenberg, Germany) and data were normalized to protein concentrations.

### Statistical analysis

Data are presented as mean ± standard error of mean (SEM). Statistical analysis was performed by the Mann–Whitney t-test, or one-way analysis of variance (ANOVA) followed by the Tukey–Kramer post hoc test (GraphPad Prism v5, San Diego, CA, USA). Values were considered significant when p < 0.05.

## Results

**Fig. 1:**
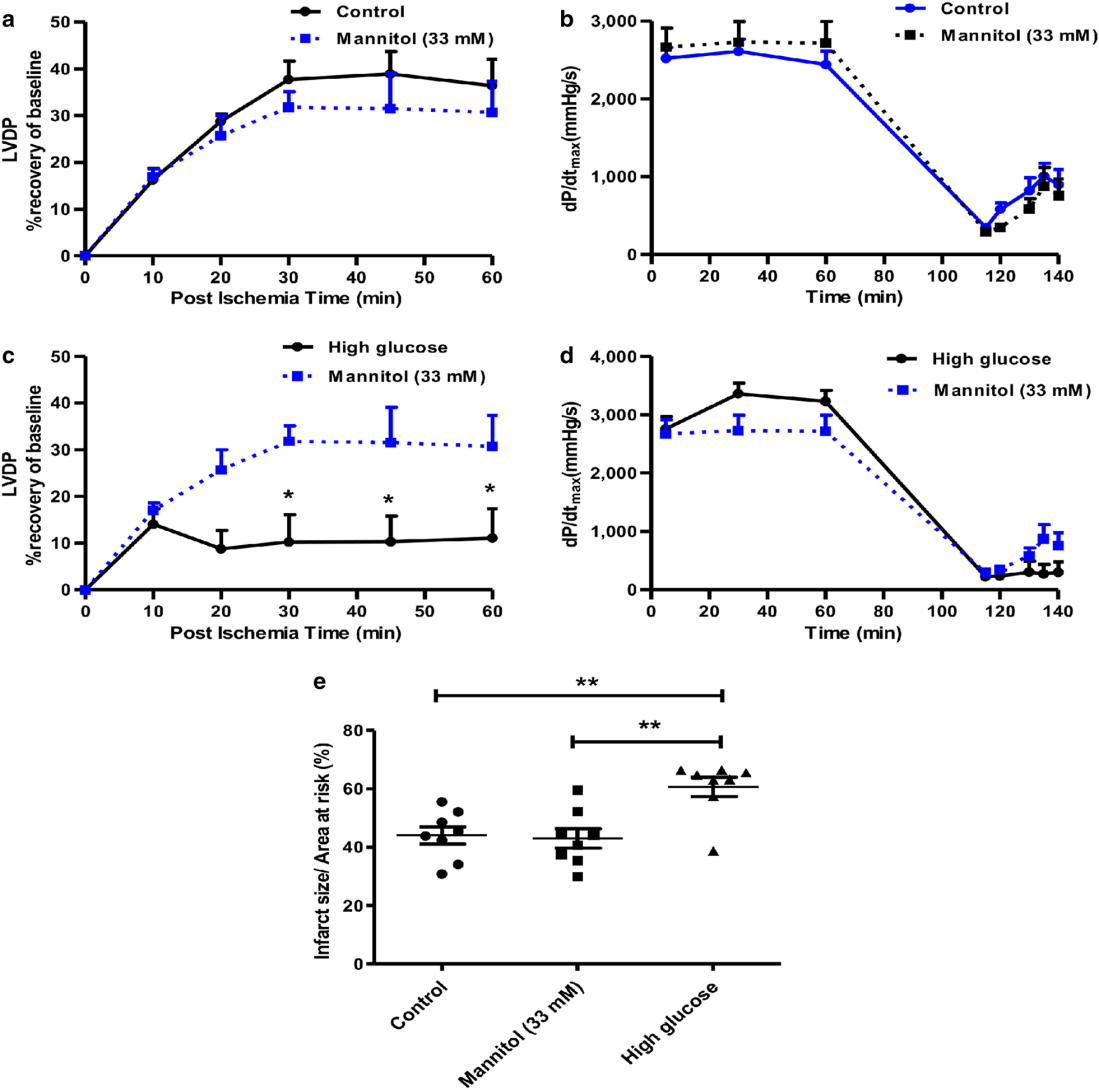
High glucose-induced cardiac contractile dysfunction independent of osmotic pressure. Isolated rat hearts were perfused under high glucose conditions (33 mM glucose) vs. controls (11 mM glucose) for 60 min and thereafter subjected to 20 min of global ischemia, followed by 60 min of reperfusion. In parallel experiments, groups were similarly perfused under hyperosmotic conditions (22 mM mannitol + 11 mM glucose; 33 mM). **a** Left ventricular developed pressure (LVDP) (% recovery) under baseline conditions vs. mannitol control, **b** maximum ventricular contraction (dP/dt_max_) under baseline glucose conditions vs. mannitol control, **c** LVDP (% recovery) under high glucose conditions vs. mannitol control, **d** dP/dt_max_ under high glucose conditions vs. mannitol control, and **e** Infarct size/area at risk (%) under baseline, high glucose and hyperosmotic conditions after subjecting rat hearts to regional ischemia as described before. Values are expressed as mean ± SEM (n = 6). *p < 0.05, **p < 0.01 vs. respective controls.

To rule out the possibility of attributing the effects of hyperglycemia on heart function to changes in osmolarity, separate perfusion experiments were completed with 11 mM glucose plus 22 mM mannitol (total molarity 33 mM). These data demonstrate no differences in LVDP recovery and dP/dt_max_ for the osmotic control group vs. hearts perfused under baseline glucose (11 mM) conditions (Fig. 1a, b). By contrast, hearts exposed to high glucose displayed lower LVDP recovery compared to the osmotic control group (p < 0.05) with no differences for dP/dt_max_ (Fig. 1c, d). High glucose perfusions also resulted in greater infarct sizes compared to both control groups (p < 0.01) (Fig. 1e).

**Fig. 2:**
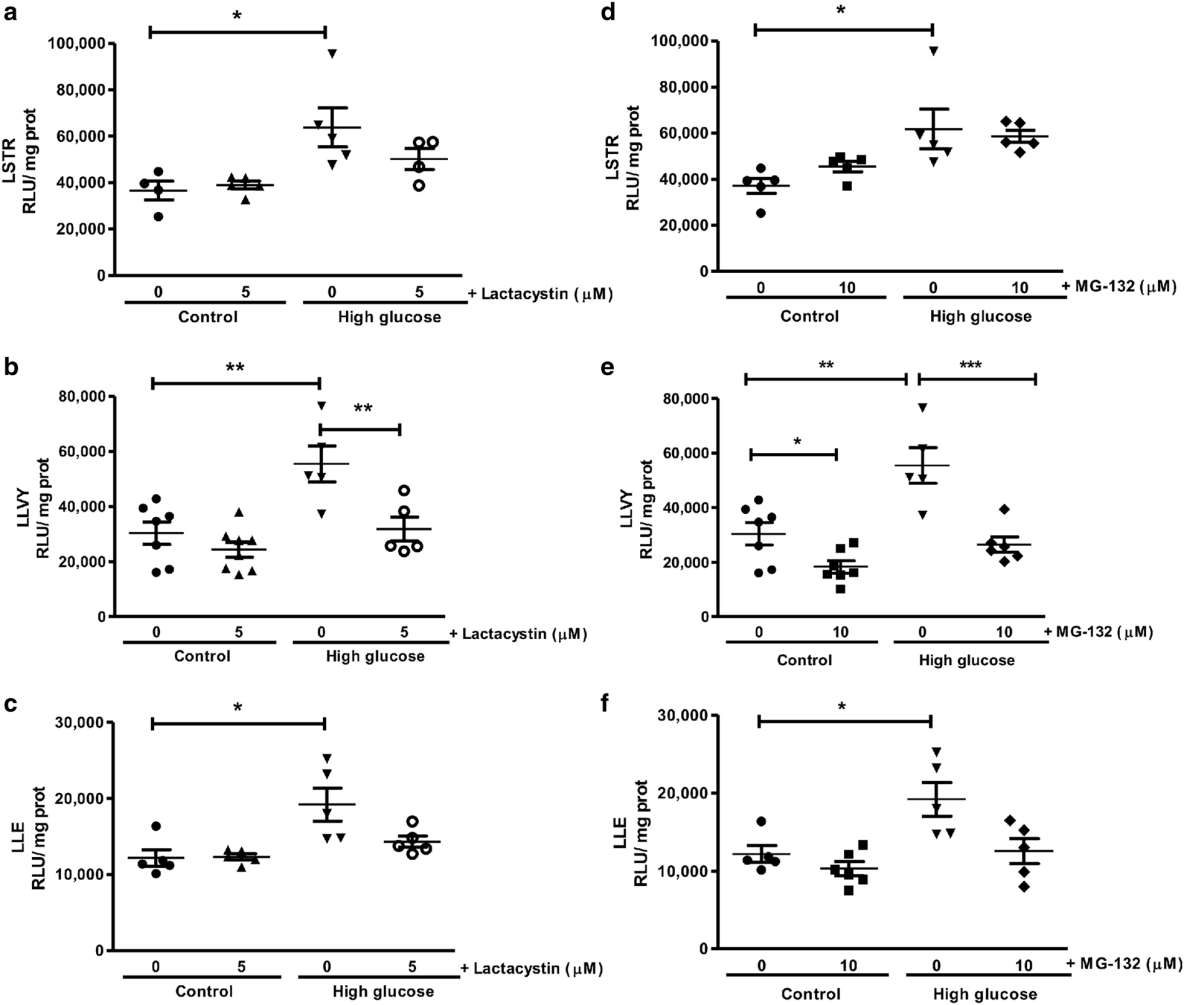
Lactacystin and MG-132 administration attenuate proteasomal activities during ischemia–reperfusion. Isolated rat hearts were perfused under high glucose (33 mM) conditions vs. controls (11 mM) and subjected to global ischemia and reperfusion as earlier described. For UPS inhibition, either 5 μM lactacystin or 10 μM MG-132 was added for the first 20 min of the reperfusion period. The respective proteasomal activities evaluated: **a**, **d** trypsin-like (LSTR), **b**, **e** chymotrypsin-like (LLVY), **c**, **f** caspase-like (LLE). Values are expressed as mean ± SEM (n = 8). *p < 0.05, **p < 0.01, ***p < 0.001 vs. respective controls.

**Fig. 3:**
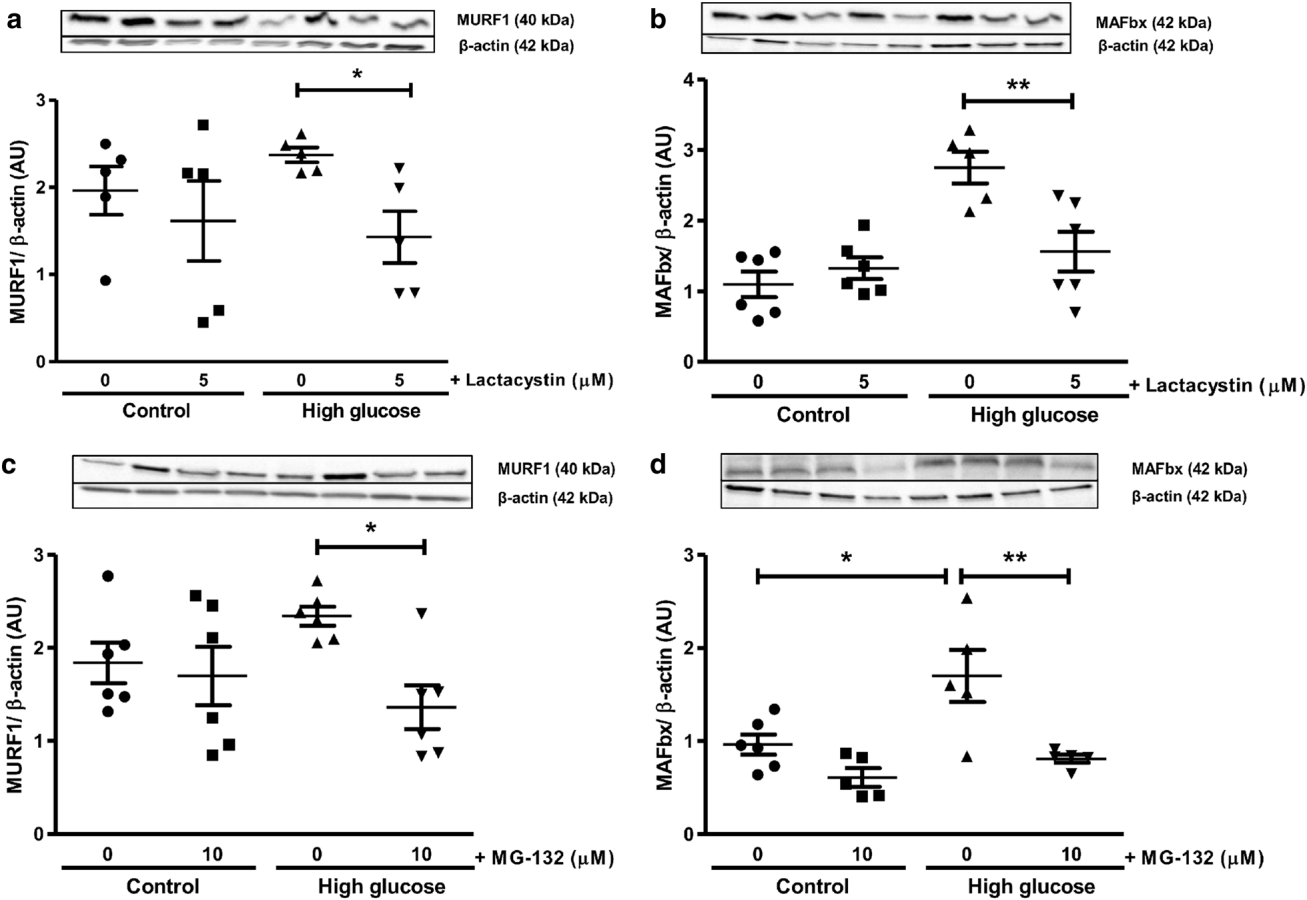
Decreased expression of E3 ubiquitin ligases during ischemia–reperfusion. Isolated rat hearts were perfused under high glucose conditions (33 mM glucose) vs. controls (11 mM glucose) for 60 min and subsequently subjected to 20 min of global ischemia, followed by 60 min of reperfusion ± either 5 μM lactacystin or 10 μM MG-132 treatment for the first 20 min of reperfusion. Western blot analysis was completed for: **a** muscle RING finger 1 (MuRF1) and **b** muscle atrophy F-box (MAFbx) with lactacystin treatment; **c** MURF1 and **d** MAFbx with MG-132 administration. We employed β-actin as loading control. Values are expressed as mean ± SEM (n ≥ 4). *p < 0.05, **p < 0.01 vs. respective controls.

We next determined the effects of high glucose exposure on post-ischemic UPS activities. Here trypsin-, chymotrypsin- and caspase-like activities were significantly upregulated (Fig. 2). The effects of lactacystin and MG-132 were determined and both compounds—as expected—significantly lowered myocardial chymotrypsin-like activity (Fig. 2). To further validate the degree of UPS inhibition we also assessed peptide levels of E3 ligases (MURF1 and MAFbx) involved in the final step of the ubiquitination process. Here MAFbx expression was significantly increased under high glucose conditions, while both lactacystin and MG-132 administration decreased MURF1 and MAFbx expression following ischemia–reperfusion under high glucose conditions (Fig. 3).

**Fig. 4:**
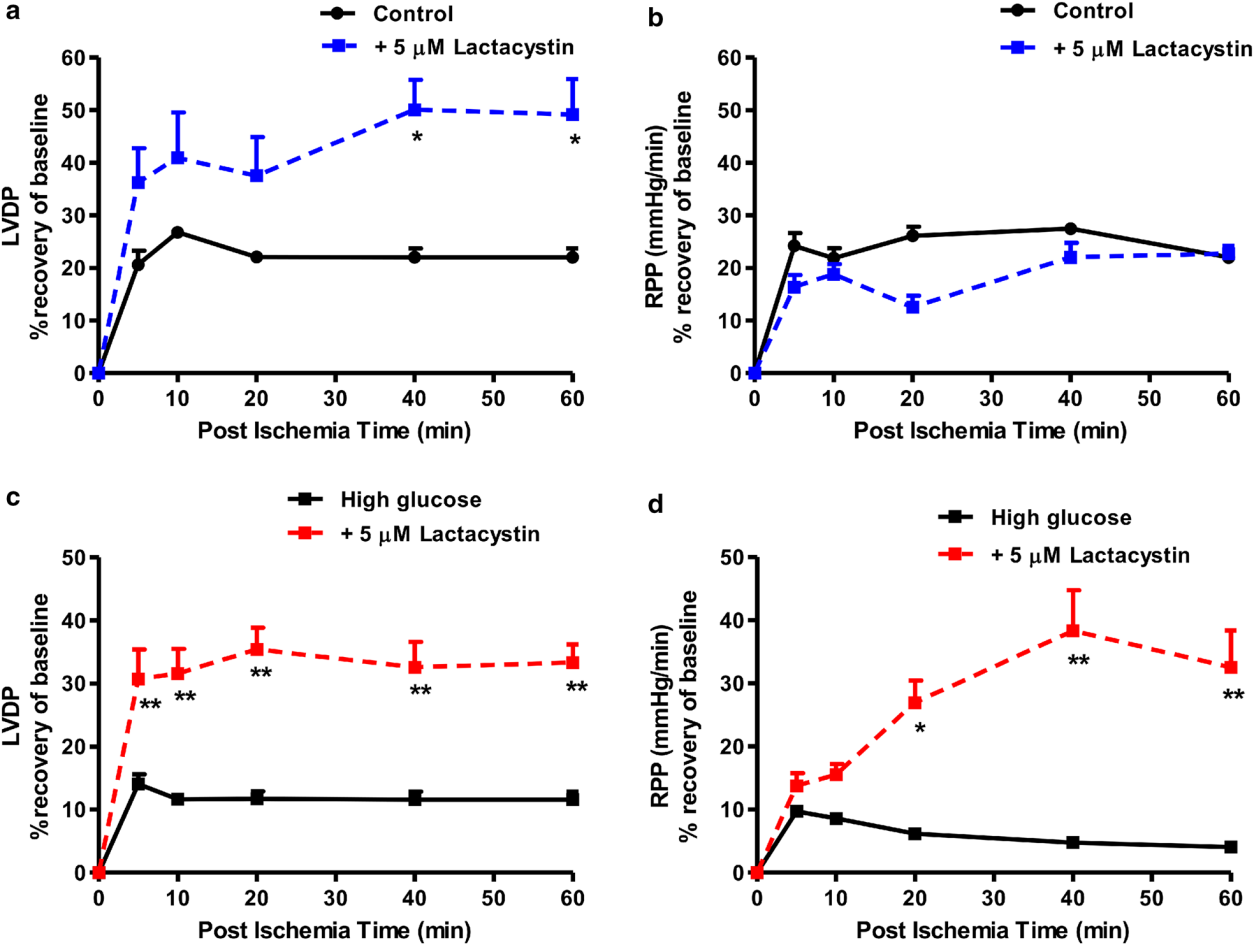
Lactacystin administration improves contractile function during ischemia–reperfusion. Isolated rat hearts were perfused under high glucose conditions (33 mM glucose) vs. controls (11 mM glucose) for 60 min and thereafter subjected to 20 min of global ischemia, followed by 60 min of reperfusion. Lactacystin (5 μM) was added to treatment groups during the first 20 min of reperfusion. **a** Left ventricular developed pressure (LVDP) (% recovery) at baseline glucose levels (11 mM), and **c** with high glucose (33 mM). Rate pressure product (RPP) (% recovery) at baseline glucose levels (**b**), and **d** under high glucose conditions. Values are expressed as mean ± SEM (n = 8).*p < 0.05, **p < 0.01 vs. respective controls.

**Fig. 5:**
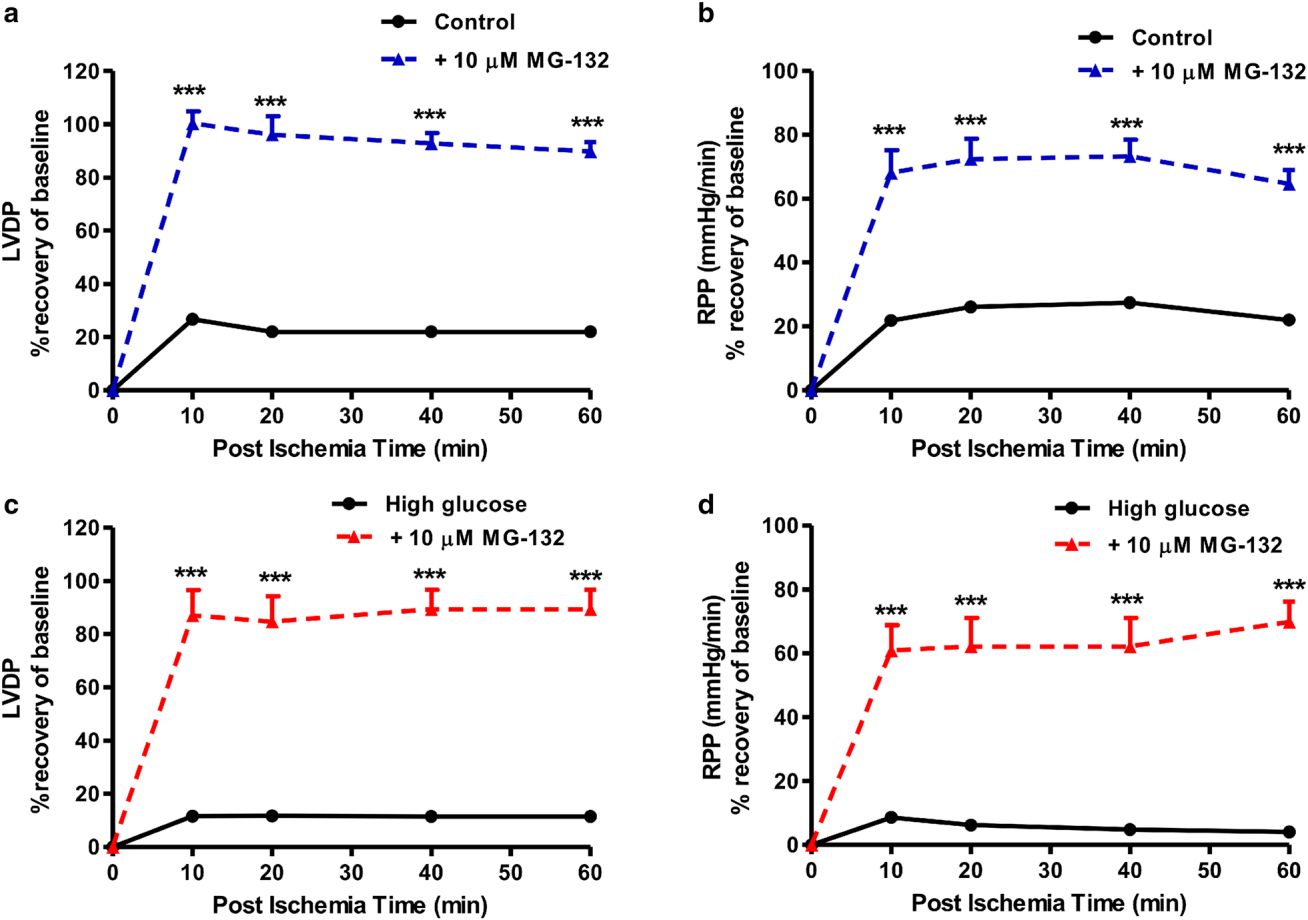
MG-132 administration improves contractile function during ischemia–reperfusion. Isolated rat hearts were perfused under high glucose conditions (33 mM glucose) vs. controls (11 mM glucose) for 60 min and thereafter subjected to 20 min of global ischemia, followed by 60 min of reperfusion. MG-132 (10 μM) was added to treatment groups during the first 20 min of reperfusion. **a** Left ventricular developed pressure (LVDP) (% recovery) at baseline glucose levels (11 mM), and **c** with high glucose (33 mM). Rate pressure product (RPP) (% recovery) at baseline glucose levels (**b**), and **d** under high glucose conditions. Values are expressed as mean ± SEM (n = 8). ***p < 0.001 vs. respective controls.

**Fig. 6:**
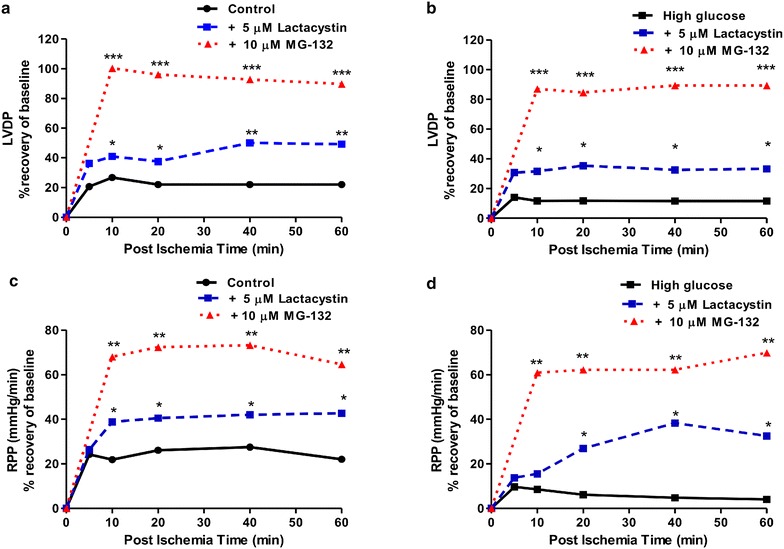
Comparative analysis of UPS inhibition on contractile function during ischemia–reperfusion. Isolated rat hearts were perfused under high glucose conditions (33 mM glucose) vs. controls (11 mM glucose) for 60 min and subsequently subjected to 20 min of global ischemia, followed by 60 min of reperfusion. Either 5 μM lactacystin or 10 μM MG-132 was added during the first 20 min of reperfusion. **a** Left ventricular developed pressure (LVDP) (% recovery) at baseline glucose levels (11 mM), and **b** with high glucose (33 mM). Rate pressure product (RPP) (% recovery) at baseline glucose levels (**c**), and **d** under high glucose conditions. Values are expressed as mean ± SEM (n = 8). *p < 0.05, **p < 0.01, ***p < 0.001 vs. respective controls.

**Fig. 7:**
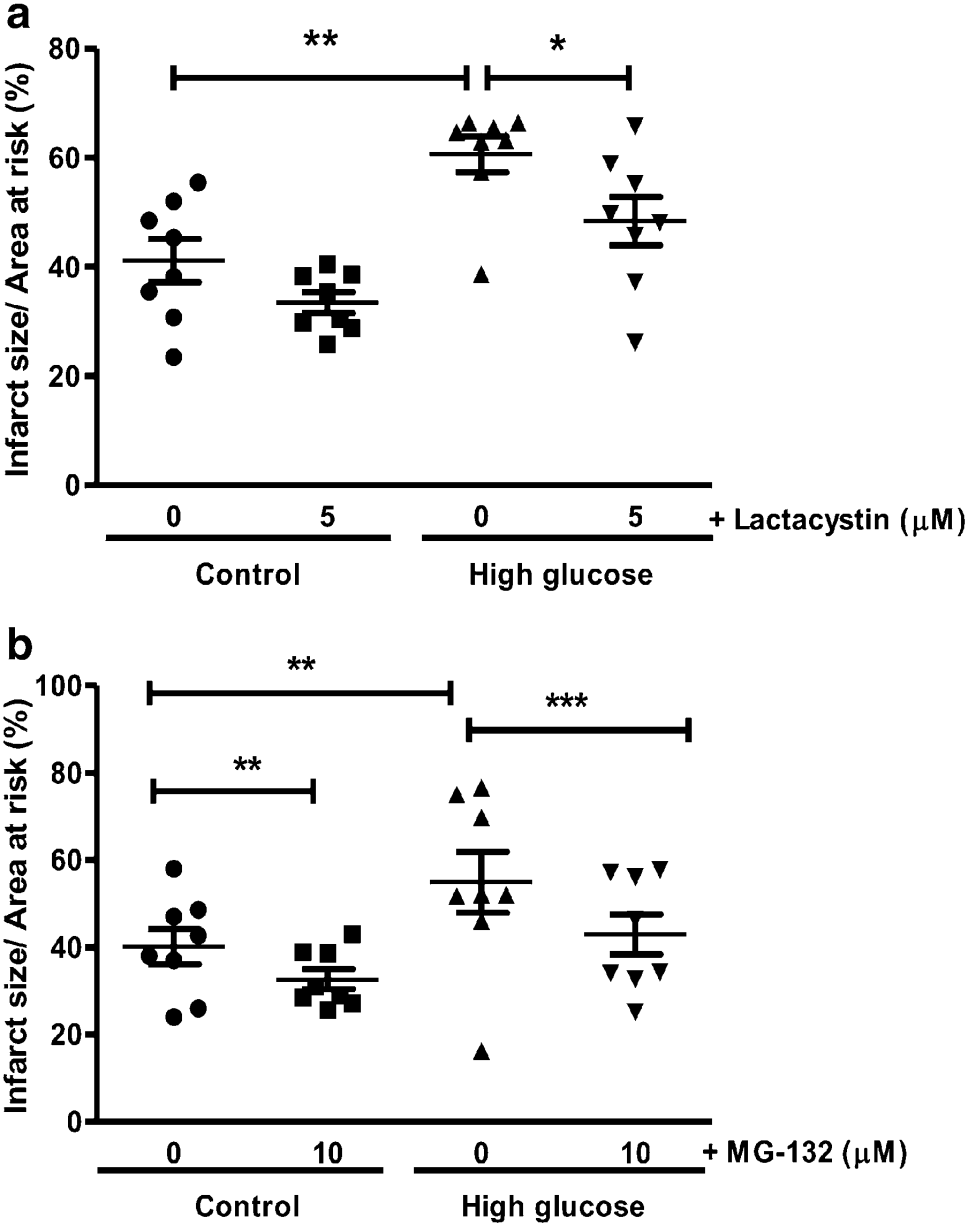
UPS inhibition attenuates infarct size. Isolated rat hearts were perfused under high glucose conditions (33 mM) vs. controls (11 mM) for 60 min and thereafter subjected to regional ischemia as earlier described. UPS inhibitors (5 μM lactacystin or 10 μM MG-132) were administered for the first 20 min of the 2-h reperfusion period. **a** Infarct size/area at risk (%) following lactacystin administration and **b** infarct size/area at risk (%) following MG-132 treatment under baseline vs. high glucose perfusion conditions. Values are expressed as mean ± SEM (n = 8). *p < 0.05, **p < 0.01, ***p < 0.001 vs. respective controls.

We next evaluated functional parameters in our experimental system and found that high glucose exposure blunted cardiac contractile function vs. matched controls following ischemia–reperfusion (Figs. 4, 5). Acute lactacystin administration during the reperfusion phase significantly improved LVDP recovery at baseline (Fig. 4a) and also under high glucose conditions (from 10 ± 2.3% to 36 ± 4%; p < 0.01 vs. untreated high glucose) (Fig. 4c). RPP data exhibited a similar trend although lactacystin had no effect under baseline glucose conditions compared to the untreated group (Fig. 4b). MG-132 elicited robust cardioprotection (LVDP, RPP) at baseline and in response to high glucose exposure (Fig. 5). Moreover, both lactacystin and MG-132 decreased end-diastolic pressure in hearts exposed to high glucose conditions, although there were no differences for the control groups (Table 1). Proteasome inhibitor treatment also enhanced coronary flow although this was only statistically significant for MG-132 (p < 0.05 vs. untreated high glucose) (Table 1). Our comparative analyses show that MG-132 administration resulted in greater cardioprotection compared to lactacystin (Fig. 6). The regional ischemia experiments further support these findings by demonstrating that lactacystin blunted the high glucose-induced increase in the infarct size/area-at-risk from 66.4 ± 3.4% to 48 ± 3.9% (p < 0.05 vs. high glucose untreated) (Fig. 7). Likewise, MG-132 treatment decreased infarct sizes under baseline and high glucose perfusion conditions. Here MG-132 significantly attenuated the infarct size/area-at-risk to 40.3 ± 2.5% and 49.7 ± 7.4% vs. 56.3 ± 2.8% and 73.5 ± 4.9% at baseline and high glucose perfusions, respectively. There were no differences in the area-at-risk for any of the experimental groups investigated (data not shown).

**Table 1:** Coronary flow, end-diastolic pressure (EDP) and heart rate (HR) under baseline vs. high glucose perfusion conditions with ischemia–reperfusion

	Coronary flow (ml/min)		EDP (mmHg)		HR (beats/min)	
	Pre-ischemia	Post-ischemia	Pre-ischemia	Post-ischemia	Pre-ischemia	Post-ischemia
Control	10 ± 2.3	8.2 ± 1.3	14 ± 0	33 ± 5	200 ± 12	240 ± 18
+5 µM lactacystin	8.4 ± 1	7.5 ± 1.5	10 ± 1	29 ± 7	235 ± 8	278 ± 14
+10 µM MG-132	12 ± 1	8 ± 2.4	11 ± 2	31 ± 2	236 ± 10	268 ± 15
High glucose	11.2 ± 1.8	6 ± 1.5	8 ± 3	65 ± 7*	347 ± 13*	244 ± 9
+5 µM lactacystin	10 ± 2.3	8.2 ± 1.3	14 ± 0	33 ± 5**	355 ± 8	248 ± 12
+10 µM MG-132	9 ± 2	11.2 ± 0.6*	15 ± 3	26 ± 7***	306 ± 10	288 ± 8

All values were recorded during the first 10 min of stabilization (“Pre-ischemia”) and at the end of reperfusion (“Post-ischemia”) and are expressed as mean ± SEM. *p < 0.05; **p < 0.01 vs. respective controls, (n = 8 per group).

**Fig. 8:**
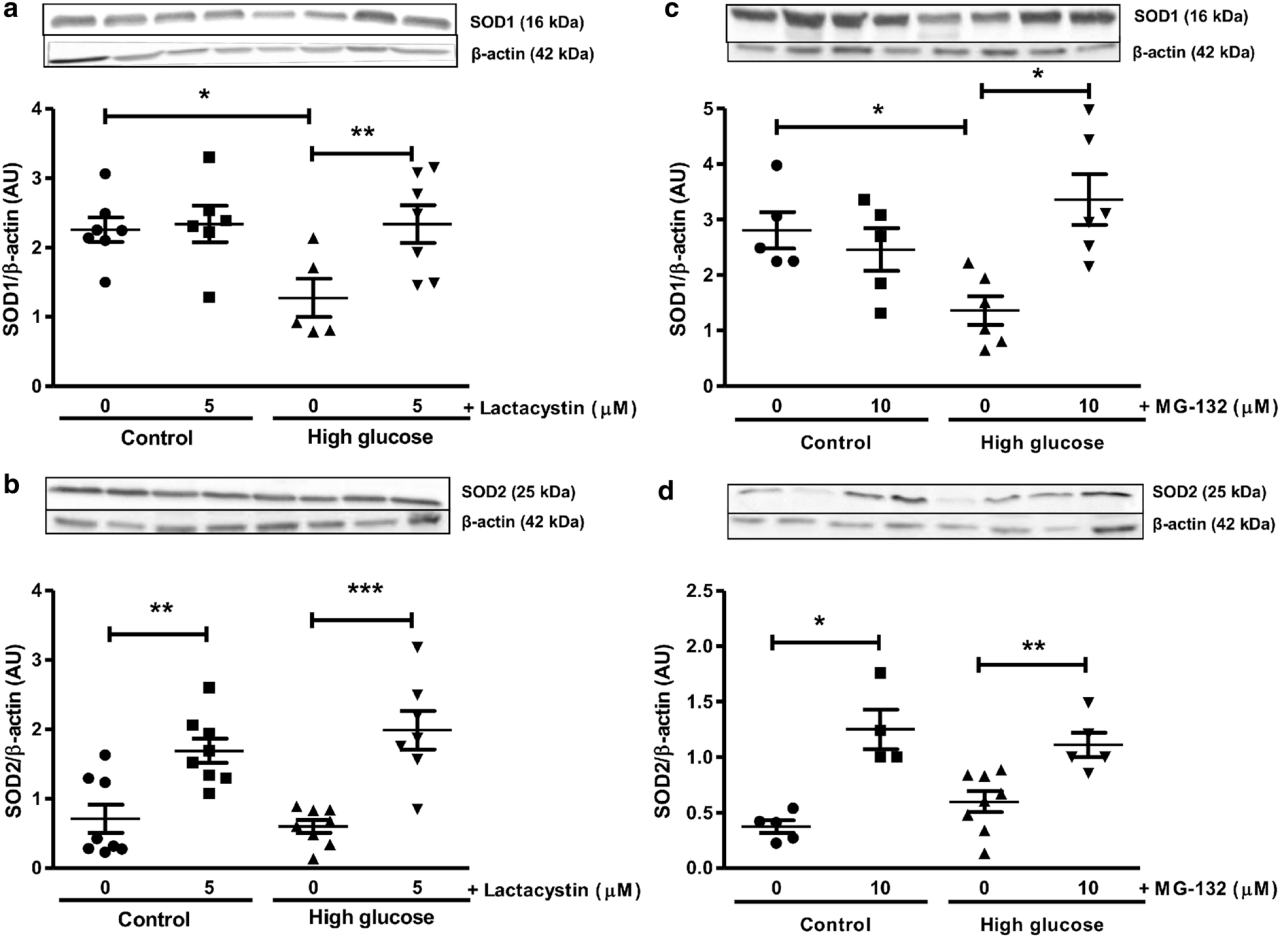
UPS inhibition enhances anti-oxidant defenses during ischemia–reperfusion. Isolated rat hearts were perfused under high glucose conditions (33 mM) vs. controls (11 mM) for 60 min and subjected to global ischemia as earlier described ± addition of 5 μM lactacystin or 10 μM MG-132 during the first 20 min of reperfusion. Western blot analysis for: **a** SOD1 and **b** SOD2 with lactacystin treatment; **c** SOD1 and **d** SOD2 with MG-132 administration. We employed β-actin as loading control. Values are expressed as mean ± SEM (n ≥ 4). *p < 0.05, **p < 0.01, ***p < 0.001 vs. respective controls.

**Fig. 9:**
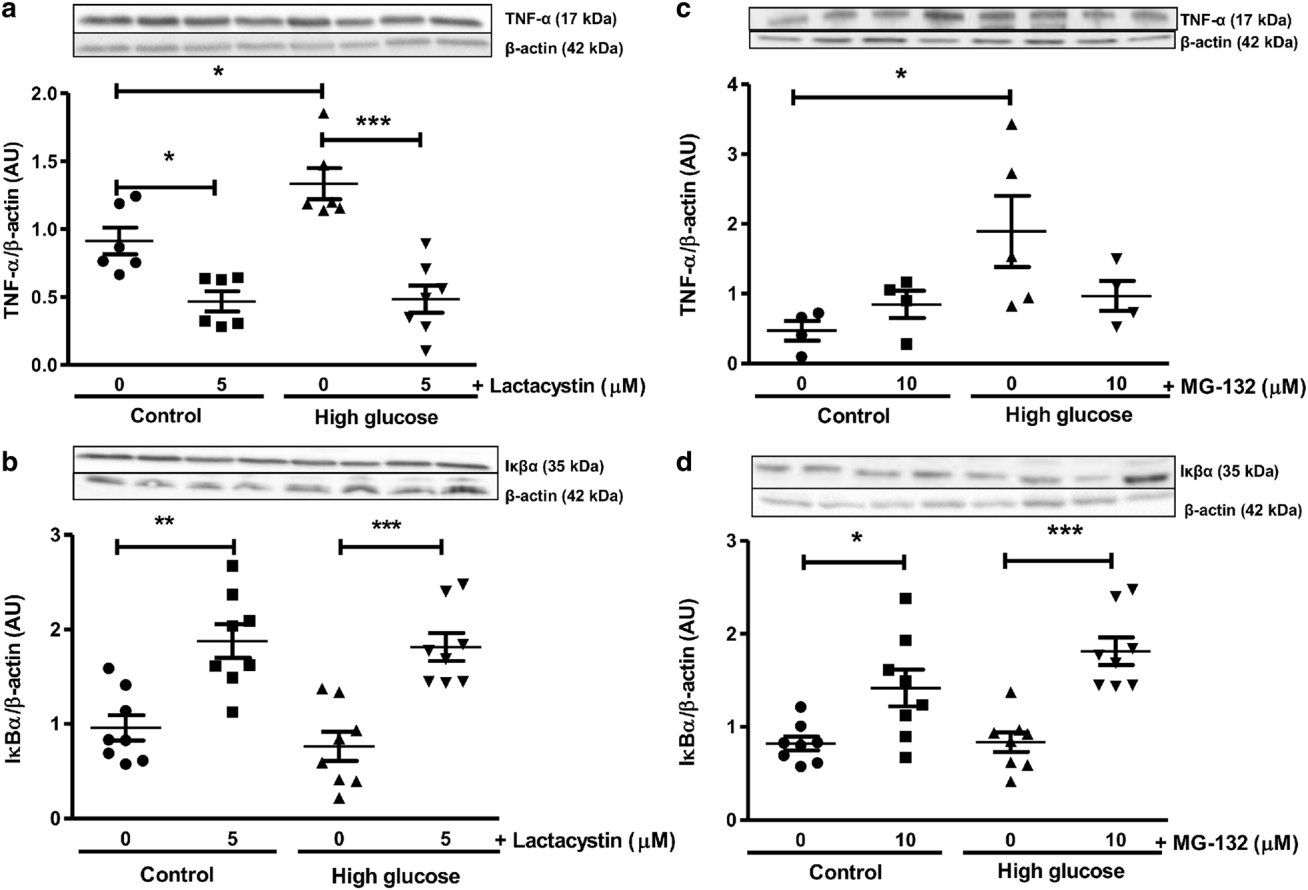
Decreased expression of inflammatory markers following UPS inhibition with ischemia–reperfusion. Isolated rat hearts were perfused under high glucose conditions (33 mM) vs. controls (11 mM) and subjected to global ischemia and reperfusion ± 5 μM lactacystin or 10 μM MG-132 treatment for the first 20 min of the reperfusion period. Western blot analysis for: **a** TNF-α and **b** IkBα with lactacystin treatment; **c** TNF-α and **d** IkBα with MG-132 administration. We employed β-actin as loading control. Values are expressed as mean ± SEM (n ≥ 4). *p < 0.05, **p < 0.01, ***p < 0.001 vs. respective controls.

**Fig. 10:**
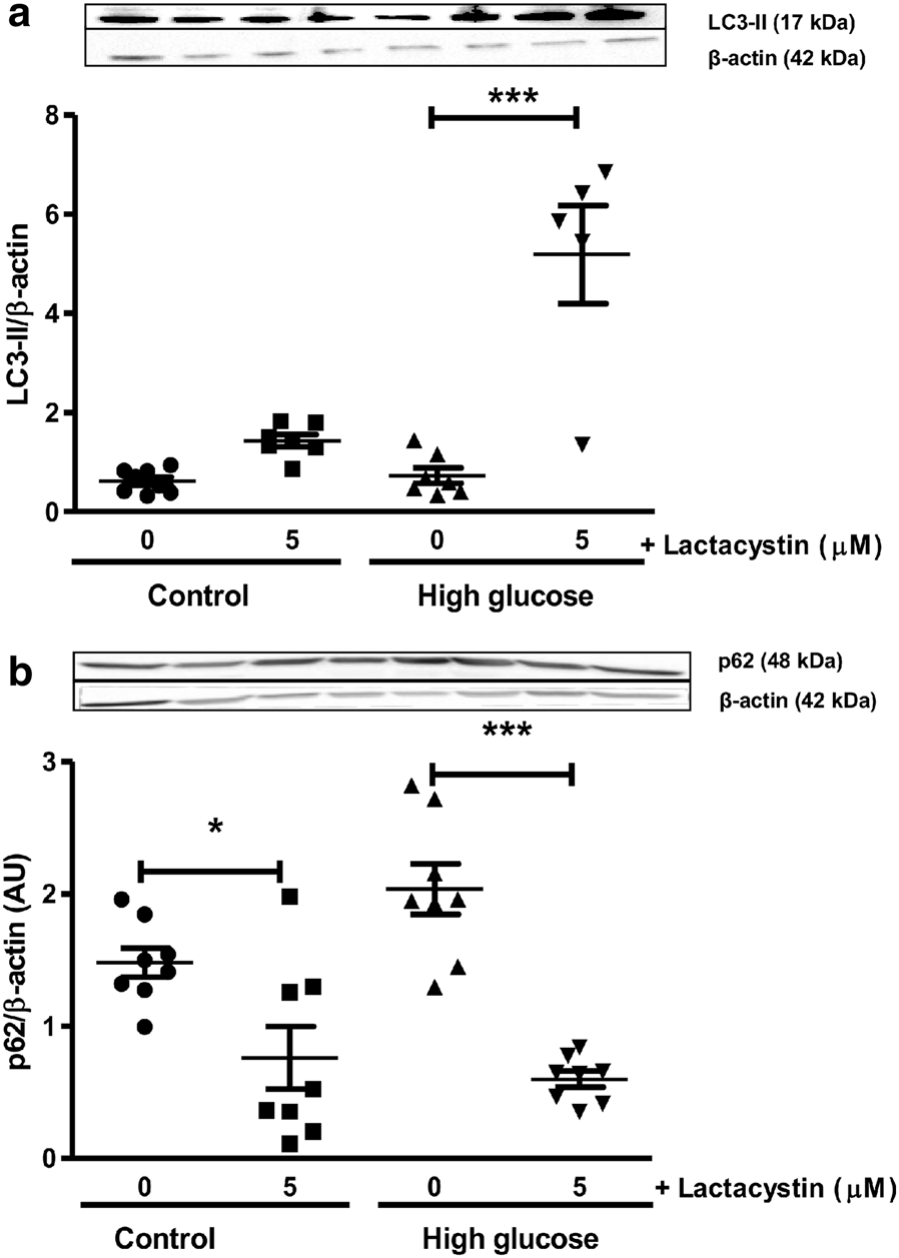
Increased expression of autophagy markers following UPS inhibition with ischemia–reperfusion. Isolated rat hearts were perfused under simulated acute hyperglycemia conditions (33 mM glucose) vs. controls (11 mM glucose) for 60 min and thereafter subjected to 20 min of global ischemia, followed by 60 min of reperfusion. 5 μM lactacystin was added for the first 20 min of reperfusion. Western blot analysis for: **a** LC3-II and **b** p62 protein expression levels are shown with β-actin as loading control. Values are expressed as mean ± SEM (n ≥ 4). *p < 0.05, ***p < 0.001 vs. respective controls.

For the current study, high glucose exposure diminished myocardial SOD1, but not SOD2 expression levels following ischemia–reperfusion (Fig. 8). Myocardial TNF-α expression was also significantly elevated while IkBα levels remained unaltered (vs. baseline glucose conditions) following ischemia–reperfusion in the presence of high glucose (Fig. 9). Due to the interplay between the UPS and autophagy we also investigated the effects of lactacystin (more specific inhibitor vs. MG-132) in this context. LC3-II protein levels were not altered following ischemia–reperfusion under high glucose conditions (Fig. 10). However, lactacystin treatment resulted in a robust elevation in cardiac LC3-II expression (p < 0.001 vs. high glucose untreated). The expression of p62 was not significantly altered in response to high glucose but was significantly increased after lactacystin administration (Fig. 10).

## Discussion

This study demonstrates that both lactacystin and MG-132 administration elicited cardioprotection during ischemia–reperfusion performed under acute high glucose conditions. Here initial experiments demonstrated a robust increase in post-ischemic myocardial UPS activities together with elevated protein expression of an E3 ligase (MAFbx). Moreover, the proteasome inhibitor doses employed and the treatment duration resulted in partial UPS impairment together with cardioprotective effects.

Previous studies found decreased proteasome function following ischemia–reperfusion, although the underlying mechanisms remain unclear (reviewed in [31]). This likely occurs due to higher free radical generation with ischemia–reperfusion, thereby leading to direct oxidative modification and subsequent attenuation of proteasome function [18, 31]. In agreement, we detected increased proteasome activities in rat hearts subjected to ischemia–reperfusion performed under baseline glucose conditions vs. pre-ischemic hearts (data not shown). However, with hyperglycemia excess oxidative stress triggers higher UPS activity to help degrade and remove damaged proteins. For example, we recently reported increased cardiac superoxide levels together with decreased SOD activity following ischemia–reperfusion performed under high glucose perfusion conditions [21, 22]. These findings are consistent with other studies demonstrating higher UPS activity in diabetic mouse hearts [15], streptozotocin-infarcted rat hearts and diabetic patients suffering from unstable angina [16]. What about the functional effects of proteasome inhibition with ischemia–reperfusion under hyperglycemic conditions? This study shows—for the first time as far as we are aware—that both lactacystin and MG-132 administration triggered cardioprotective effects with ischemia–reperfusion performed under such conditions. This is in agreement with previous findings showing that high glucose levels trigger myocardial oxidative stress and apoptosis in parallel with impaired contractile function [22].

The effects observed under baseline glucose conditions are consistent with earlier research establishing that proteasome inhibition results in cardioprotection with ischemia–reperfusion [17, 32–34]. Although both beneficial and detrimental effects were observed with proteasome inhibition, the collective data indicate that cardioprotection with ischemia–reperfusion occurs only with acute drug administration [31]. Moreover, the outcomes of UPS inhibition are strongly dose- and time-dependent with lower, non-toxic doses likely upregulating anti-oxidant defenses and exerting anti-inflammatory effects, while higher doses and chronic treatment may result in damaging consequences on protein turnover and cell death [31]. This may also depend on distinct intracellular sub-compartments, with some suggesting that beneficial effects may be due to the inhibition of proteasomal activity within the non-cardiomyocyte compartment where it may exert anti-inflammatory effects [35]. In support, others found that moderate cardiomyocyte-restricted proteasome inhibition exacerbated ischemia–reperfusion injury in mouse hearts [36]. Further studies are, however, required to investigate these intriguing possibilities.

The comparative analyses performed establish that MG-132 administration lead to greater cardioprotection compared to lactacystin. We are unclear regarding the underlying mechanism(s) whereby MG-132 resulted in such a striking improvement in cardiac function. As indicated before, proteasome inhibitor dose and treatment duration are crucial parameters that may affect functional outcomes and we therefore cannot exclude the possibility that lower lactacystin dosages could potentially result in enhanced cardioprotection. Lactacystin is also considered a more specific inhibitor than MG-132 and it is therefore possible that the superiority of MG-132 may be due to non-UPS-related effects. For example, MG-132 can exert additional effects on calpains and cathepsins [37] and also protect cardiomyocytes from injury through the inhibition of NF-kB and downregulation of transforming growth factor beta 1 (TGFβ1) [38]. As MG-132 significantly augmented coronary flow, it is possible that it may also exert advantageous effects on cardiac endothelial cells. In support, an in vitro study found that low-dose proteasome inhibition upregulated endothelial nitric oxide synthase expression and activity together with improved endothelial function [39]. This may therefore lead to increased oxygen supply to the myocardium resulting in improved post-ischemic function [40].

To gain a better understanding of the mechanisms whereby proteasome inhibitors elicit cardioprotection, we assessed whether it exhibits anti-oxidant and/or anti-inflammatory properties in our experimental system. It is well established that myocardial ischemia–reperfusion is associated with increased oxidative stress resulting in cell death [41, 42]. For example, we recently reported that several markers of myocardial oxidative stress were significantly increased in our experimental model [21, 22]. Of note, high glucose availability leads to oxidative stress due to an imbalance in terms of free radical production and intracellular anti-oxidant defenses [43–45]. For the current study high glucose exposure diminished myocardial SOD1, but not SOD2 expression levels following ischemia–reperfusion. Both lactacystin and MG-132 administration resulted in higher cardiac SOD1 and SOD2 protein levels and this is likely a key mechanism to detoxify increased free radical levels. Although we are unclear regarding the transcriptional modulators implicated in this process, the basic leucine zipper transcription factor, nuclear factor-erythroid 2-related factor 2 (Nrf2), is a strong candidate as it is a known UPS target [46, 47]. It is likely that UPS inhibition in our experimental system attenuates Nrf2 ubiquitination and subsequent degradation, thereby increasing its stability and translocation to the nucleus for activation of target anti-oxidant genes [48–50].

The effects of proteasome inhibition on myocardial inflammation were investigated by evaluating TNF-α peptide levels and NFkβ activation. For the latter, we determined myocardial IkBα levels—a UPS target—that when bound to NFκβ in the cytosol prevents its translocation to the nucleus and the subsequent activation of target genes such as TNF-α. Here TNF-α expression was significantly elevated while IkBα levels remained unaltered following ischemia–reperfusion in the presence of high glucose. Lactacystin administration blunted the TNF-α spike under baseline and high glucose conditions, while this effect was not observed with MG-132. However, IkBα levels were significantly higher following lactacystin and MG-132 administration. As lactacystin is a more potent proteasome inhibitor than MG-132, this suggests that its cardioprotective effects are more strongly linked to the attenuation of UPS-mediated induction of inflammatory pathways. In support, a disproportionately elevated UPS can lead to NFκβ activation, thereby resulting in downstream transcriptional effects and the establishment of a pro-inflammatory milieu with hyperglycemia and/or ischemia [16]. Thus these data establish that the strong pro-inflammatory effect triggered in response to ischemia–reperfusion and hyperglycemia can be blunted by partial proteasome inhibition.

Due to the interplay between the UPS and autophagy we also investigated the effects of lactacystin (more specific inhibitor vs. MG-132) in this context. LC3-II levels increased while there was also a significant decrease in p62 expression, indicating greater autophagic flux. Here p62 can shuttle ubiquitinated proteins not removed by the UPS to the autophagic clearance system [51], thus serving as an adapter that is able to interact with both the UPS and autophagic machinery to eventually help with the degradation of misfolded and damaged proteins [52]. These data are consistent with others demonstrating that low dose proteasome inhibitor treatment (reducing chymotrypsin-like activity to ~46%) increased myocardial autophagy [53]. Lower p62 levels would indicate greater clearance and uptake of damaged proteins by cardiac autophagosomes. Thus with UPS inhibition there is an upregulation of autophagy following ischemia–reperfusion under high glucose conditions, likely an adaptive mechanism that assists with the removal of misfolded and damaged proteins. This is in agreement with others reporting that experimentally-induced upregulation of autophagy was linked to improved heart function in diabetic mice [54].

### Limitations of study

For the proteasome activity assays we did not employ proteasome inhibitors and the data here presented may also contain contributions from non-proteasomal proteases. Thus it is possible that the degree of proteasomal inhibition could be an underestimation, although this should not affect our relative comparisons.

## Conclusions

This study established that partial proteasome inhibition elicits cardioprotection in hearts exposed to ischemia–reperfusion with acute simulated hyperglycemia. Our findings also show three major protective effects, i.e. (a) enhancing myocardial anti-oxidant defences, (b) attenuating inflammation, and (c) increasing the autophagic response. These data are encouraging as it could lead to the development of novel therapeutic interventions that may help treat individuals burdened with hyperglycemia and suffering associated cardiovascular complications such as an AMI.

## Abbreviations

µg: microgram
µl: microliter
µM: micromolar
°C: degree Celsius
AMI: acute myocardial infarction
ANOVA: one way analysis of variance
CA: California
CaCl_2_: calcium chloride
CMRG: Cardio-Metabolic Research Group
CO_2_: carbon dioxide
dP/dt_max_: maximum velocity of contraction
EDP: end diastolic pressure
*g*: gravitational acceleration
g: gram
HR: heart rate
h: hour
IκBα: inhibitor of nuclear factor kappa-B kinase subunit alpha
i.p.: intraperitoneal
Inc.: incorporation
KH_2_PO_4_: potassium dihydrogen phosphate
LC3-II: microtubule‐associated protein 1 light chain 3 II
LLE: caspase-like proteasome activity
LLVY: chymotrypsin-like proteasome activity
LSTR: trypsin-like proteasome activity
LVDP: left ventricular developed pressure
MA: Massachusetts
MAFbx: muscle atrophy F-box
mg/kg: milligrams per kilogram
MgCl_2_: magnesium chloride
MgSO_4_: magnesium sulphate
min: minute(s)
mmHg: millimetres of mercury
mM: millimolar
MO: Missouri
MURF1: muscle RING finger1
n: number
NaCl: sodium chloride
NaHCO_3_: sodium hydrogen carbonate
NFkβ: nuclear factor kappa beta
NIH: National Institutes of Health
Nrf2: basic leucine zipper transcription factor, nuclear factor-erythroid 2-related factor 2
NSW: New South Wales
O_2_: oxygen
RIPA: radioimmunoprecipitation assay
RPP: rate pressure product
SDS-PAGE: sodium dodecyl sulfate polyacrylamide gel electrophoresis
SEM: standard error of the mean
SOD1/2: superoxide dismutase 1/2
TGFβ1: transforming growth factor beta 1
TNF-α: tumor necrosis factor-alpha
UPS: ubiquitin proteasome system
USA: United States of America
vs.: versus
WI: Wisconsin
